# POLIS: a rapid and replicable screening tool to improve risk stratification of potential false-negative COVID-19 patients

**DOI:** 10.1136/postgradmedj-2020-138190

**Published:** 2020-07-24

**Authors:** Eng Chuan Foo, Anli Yue Zhou, Oliver Lily

**Affiliations:** Leeds Centre for Neurosciences, Leeds Teaching Hospitals NHS Trust, Leeds, UK; The Centre for Occupational and Environmental Health, The University of Manchester, Manchester, UK; Leeds Centre for Neurosciences, Leeds Teaching Hospitals NHS Trust, Leeds, UK

The SARS-CoV-2 (COVID-19) pandemic at the time of this writing has infected 233, 151 people in the UK with 33 614 deaths.^1^ Current UK guidance for COVID-19 virus testing states that the preferred screening/testing technique is molecular diagnosis using a real-time RT-PCR (RdRp gene) assay.^2^

Initial COVID-19 testing likely underestimates the number of actual COVID-19 cases in hospitals as false-negative rates have been to found to be between 10% and 30%.^3 4^ This could potentially impact on clinical decisions such as ward designation, for example, isolation rooms, COVID-19 wards, use of appropriate personal protective equipment (PPE)^5^ as well as workforce allocation. There is, therefore, a need to develop a tool using readily available information in the acute setting to risk stratify suspected COVID-19 patients.

A literature search was undertaken using keywords such as symptoms, presentation, investigations and COVID-19 in PubMed to recognise clinical factors that are important in identifying COVID-19 in patients. We did not identify any similar tools that were available in the current available literature. Using evidence from current literature, clinical factors and considering the clinical information available to the frontline medical staff at our hospital, a simple tool (POLIS) was devised:^3^  ^6^  ^8^

- **P**rocalcitonin (<0.5 ng/mL) with raised C-reactive protein (CRP) (1 point).
- **O**xygen saturation <93% on air despite previously healthy lungs (hypoxia) (1 point).
- **L**ymphopenia with normal white blood count (1 point).
- **I**maging: Chest X-ray suspicious of COVID-19 (1 point).
- **S**ymptoms: suggestive of COVID-19 infection (eg, fever, persistent cough/dry cough, lethargy, anosmia/ageusia, myalgia) (1 point).

This tool was applied to all suspected COVID-19 patients admitted to our department over a 5-week period, and patients were stratified into three groups that guided further management:

- **Score 0–1**: Unlikely COVID-19 infection. Management on standard acute assessment wards and to use PPE recommended by Public Health England (PHE) guidance for working in an emergency department/acute assessment area with possible or confirmed case(s)—direct patient care (within 2 m).^5^
- **Score 2**: Possible COVID-19 infection. Manage on higher risk acute care area as a suspected false-negative patient, consider retesting for COVID-19 and consider further diagnostics, for example, CT scan. Use PPE recommended by PHE guidance for working in an emergency department/acute assessment area with possible or confirmed case(s)—direct patient care (within 2 m).^5^
- **Score 3 or above:** Clinically highly likely COVID-19 infection, manages in a higher risk acute care area as a positive COVID-19 patient. Retest for COVID-19. PPE recommended by PHE guidance for working in a higher risk acute care area with possible or confirmed case(s).^5^

In total, 84 patients were admitted to our department as suspected COVID-19 cases over a 5-week period between April and May 2020. Of these, 34 (40%) patients tested positive on initial testing. In the 50 patients who tested negative, 26 had a POLIS score of 0–1. In the remaining 24 patients, 11 had a POLIS score of 2, and 13 had a POLIS score of ≥3.

All 24 patients with POLIS score 2 and above were retested for COVID-19. Of these, 16 (68%, (POLIS score 2=6, POLIS score ≥3=10)) were positive for COVID-19 on the second test. In total, 55% of patients with POLIS score 2 and 77% ofpatients with POLIS score ≥3 tested positive for COVID-19 on retesting. Our results showed a false-negative rate of 32% for the initial test on COVID-19-suspected patients.

Our preliminary results suggest that false-negative results from initial COVID-19 tests are not uncommon, and almost one-third of our negative cases were found to be positive on retesting. Therefore, relying on a single COVID-19 test could lead to potential mismanagement of false-negative cases, and risk infection spread to potentially vulnerable inpatients as well as healthcare staff. We would recommend that patients with a high score are treated as high risk even if repeat testing is negative, as sensitivity of testing reduces over time, leading to further false-negative results.^9^

The POLIS tool can enable frontline medical staff not only to triangulate data that are readily available but also to appropriately manage patients using a timely, replicable method. This helps facilitate appropriate ward designation, resource usage such as PPE as well as workforce allocation when a false-negative COVID-19 test is suspected. In cases where the COVID-19 test result is pending, this is also a useful tool to guide appropriate ward designation and ensure smooth patient flow for suspected patients referred from the emergency department. We appreciate our sample size is relatively small, and therefore more data are required to make more robust conclusions.

Previous risk management suggestions from the USA include a comprehensive range of tests and investigations^3^; however, in a healthcare system that is already under significant pressure, replicable and rapid tools such as POLIS are useful to help risk stratification, facilitate efficient patient flow and guide appropriate resource usage, particularly in a busy acute setting.
